# Association between sleep duration and musculoskeletal pain: The Korea National Health and Nutrition Examination Survey 2010–2015

**DOI:** 10.1097/MD.0000000000013656

**Published:** 2018-12-14

**Authors:** Min Young Chun, Bum-Joo Cho, Sang Ho Yoo, Bumjo Oh, Ju-Seop Kang, Cholog Yeon

**Affiliations:** a Department of Global Medical Science, Sungshin Women's University; b Department of Pharmacology & Clinical Pharmacology Lab, College of Medicine, Hanyang University, Seoul; c Department of Ophthalmology, Hallym University College of Medicine, Chuncheon Sacred Heart Hospital, Chuncheon; d Department of Medical Humanities and Ethics, Hanyang University College of Medicine; e Department of Family Medicine, SMG - SNU Boramae Medical Center, Seoul, Republic of Korea; f College of Medicine, American University of Antigua, Antigua and Barbuda, USA.

**Keywords:** joint pain, Korean population, low back pain, musculoskeletal pain, sleep duration

## Abstract

Both extremely long and short sleep durations have been associated with increased risk of numerous health problems. This study examined the association between self-reported sleep duration and reporting of musculoskeletal pain in the adult Korean population.

This study included data from 17,108 adults aged ≥50 years, obtained from the Korea National Health and Nutrition Examination Survey 2010–2012 and 2013–2015. Self-reported daily hours slept and the presence of musculoskeletal pain in knee joint, hip joint, or low back were examined. Patients were stratified into 5 groups by their sleep duration: ≤5, 6, 7, 8, or ≥9 h. Multivariate logistic regression analysis was performed, adjusting for covariates including age, sex, marital status, smoking, alcohol use, family income level, education, physical exercise, body mass index (BMI), and stress level.

A U-shaped relationship was observed between the length of sleep duration and the presence of musculoskeletal pain. After adjusting for covariates, sleep duration of ≤5 h or ≥9 h was significantly associated with musculoskeletal pain experienced for more than 30 days over a 3-month period. We also found that the presence of multi-site musculoskeletal pain was significantly higher among those who slept for ≤5 h or ≥9 h than in those who slept for 7 h.

These findings suggest that either short or long sleep duration is associated with musculoskeletal pain among Korean adults.

## 1 Introduction

Musculoskeletal (MSK) pain is highly prevalent in old age and can be disabling to sufferers, resulting in significant economic burden and a detrimental impact on quality of life.^[1–5]^ Given the negative impact of pain, finding risk factors for MSK pain or the factors augmenting adverse outcomes has been important. Of special interest are modifiable factors such as intervention target; sleep might be one of the adjustable risk factors.

In many studies, sleep deprivation has been reported to have adverse effects on clinical outcomes in patients with MSK pain, as it worsens pain levels, psychological health status, and physical functionality.^[6–8]^ Various clinical researches have examined the association between sleep and musculoskeletal pain.^[2,6–10]^ Sleep disturbance is also related to several systemic diseases, such as type 2 diabetes, coronary heart disease, and stroke, and ultimately an increase in early mortality.^[11–13]^ Of note, a recent study found that improvement in sleep problems can lead to better treatment outcomes in chronic pain patients.^[9]^ Although the underlying mechanisms of the relationship are unclear, there might be a possibility of a link between sleep duration and pain.

Nevertheless, large epidemiologic studies evaluating the correlation between sleep duration and different types of MSK pain, including knee joint pain, hip joint pain, and back pain, particularly adjusting for sociodemographic factors and comorbid conditions, are very limited. Therefore, this study aimed to investigate the association between self-reported sleep duration and MSK pain in the Korean adult population, using a nationally representative dataset obtained from the Korea National Health and Nutrition Examination Survey (KNHANES). We hypothesized that sleep duration would contribute significantly to reports of MSK pain and performed multivariate analyses on this association.

## 2 Materials and methods

### 2.1 Study population

**Figure 1:**
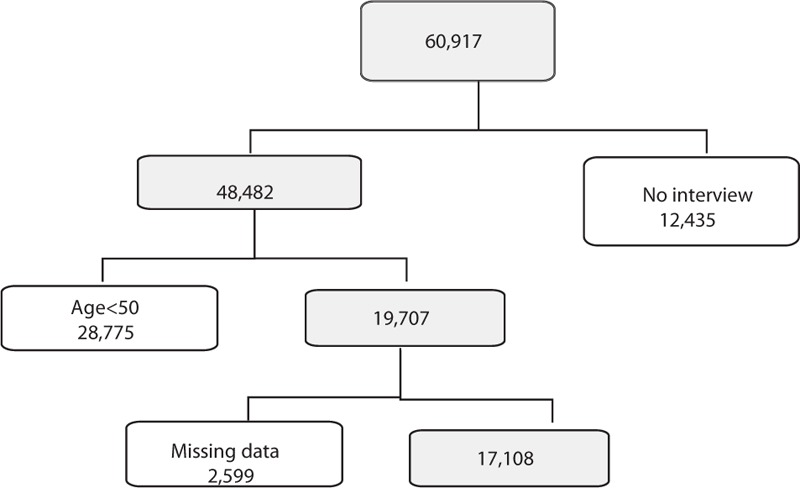
Flowchart showing inclusion and exclusion of subjects according to study criteria.

The data for this study were acquired from the fifth (2010–2012) and sixth (2013–2015) KNHANES that were conducted by the Korea Center for Disease Control and Prevention. The survey is a nationwide representative study that used stratified, multistage probability sampling to select household units. The samples were decided by the household registries of the 2005 National Census Registry. Among the total of 60,917 subjects (Fig. 1), 25,534 and 22,948 participants completed the 2010–2012 and 2013–2015 KNHANES assessments, which had average response rates of 80.8% and 78.3%, respectively. Of the 48,482 people who participated in the 2010–2015 survey, 28,775 subjects aged <50 years and 2599 with missing information for variables analyzed in this study were excluded, leaving 17,108 adults aged ≥50 years for inclusion in the final analysis.

The KNHANES comprises a physical health examination and 3 self-reported questionnaires (health behavior, health interview, and nutrition).^[14]^ The health interview survey was conducted using self-administered structured questionnaires to obtain information on sociodemographic characteristics, health status, health service use, and health behaviors. The survey information was collected by face-to-face interview following agreement from the individual, and a physical health examination was performed. All study participants provided written informed consent, and the institutional review board of the Korea Center for Disease Control and Prevention (Approval Numbers: 2010-02CON-21-C, 2011-02CON-06-C, 2012-01EXP-01-2C, 2013-07CON-03-4C,2013-12EXP-03-5C) and the Institutional Review Board of Sungshin Women's University (Institutional Review Board No. SSWUIRB 2018-016) approved the study protocol.

### 2.2 Measurements

#### 2.2.1 Sleep-duration assessment

In this study, habitual sleep time was assessed based on self-reported data in response to the question “How many hours do you usually sleep per day on average?” The answer for sleep duration was obtained as a numerical value of hours. Responses ranged from 0 to 20 h and were categorized into 5 groups based on the mean sleep duration (6.6 h): ≤5, 6, 7, 8, and ≥9 h.

#### 2.2.2 Musculoskeletal pain assessment

Musculoskeletal pain was defined as pain in the following 3 anatomical areas: knee, hip, and low part of the back. Knee pain was defined with a “Yes” response to the question “Have you had knee pain for more than 30 days in the past 3 months?” Hip pain and low back pain were respectively defined as the case in which the response was “Yes” to the question “Have you had hip pain for more than 30 days in the past 3 months?” and the case in which the response was “Yes” to the question “Have you had back pain for more than 30 days in the past 3 months?”

#### 2.2.3 Demographic and health behavior variables

Demographic and health behavior variables included in this analysis were age, sex, marital status, monthly family income, education, occupation, smoking status, alcohol use, and physical activity. Data were obtained from the health interview survey on age, sex (male/female), marital status (married/single/separated), education (none/elementary school/middle school/high school/college or above) and occupation (white collar/blue collar/unpaid employment). Family income was adjusted for the number of family members and divided into four quartiles. Frequency of alcohol use was classified into 2 groups according to the frequency of alcohol consumption per month during the 1-year period before the interview (≥4 times/month, <4 times/month). Smoking status was classified as current smoker, ex-smoker, or non-smoker. Regular physical activity was categorized according to the frequency of performance of moderate-intensity activity (at least 10 min/day per week): never, 1–3 days per month, 4–6 days per month, every day.

#### 2.2.4 Health status variables

The level of perceived stress was assessed based on self-reported data in response to the question “How much stress do you usually feel?” The responses were categorized into low, high, or very high. Trained examiners measured participants’ height (cm) and weight (kg), which was used to calculate body mass index (BMI). BMI is a crude population measure of obesity, which is calculated by dividing an individual's weight by the square of their height (kg/m^2^). Because MSK pain is affected by the pathology of each joint, the active illnesses osteoarthritis and rheumatoid arthritis were also included in our analysis. The data were obtained from the health interview (yes/no).

### 2.3 Statistical analysis

We performed the independent *t* test and chi-squared tests to analyze differences according to the presence of MSK pain symptoms (knee joint pain, hip joint pain, low back pain). Nominal variables were expressed as frequencies and percentages±standard errors and continuous variables were expressed as means ± standard errors. In addition, after participants were stratified into 5 groups based on sleep duration, chi-square tests were used. We applied a multivariate logistic regression analysis to determine the association between sleep duration and musculoskeletal pain, after controlling for other covariates.

The included covariates were age, sex, marital status, family income, education, occupation, smoking, alcohol use, physical activity, BMI, and stress levels, which were examined to determine which had a significant relationship with sleep duration or MSK pain. Model 1 was unadjusted, model 2 was adjusted for demographics (age, sex, marital status, family income, education, occupation), model 3 was adjusted for demographics and health behavior factors (smoking, alcohol use, physical activity) and model 4 was adjusted for demographics, health behavior factors, and health status factors (BMI, stress levels). Sleep duration was categorized into 5 groups: ≤5, 6, 7 (reference), 8, and ≥9 h. The adjusted odds ratios (ORs) and the corresponding 95% confidence intervals (CIs) were obtained, relating each category of sleep duration to each musculoskeletal pain. Furthermore, after participants were divided into 5 groups based on the number of sites where they experienced musculoskeletal pain, chi-square tests were performed to assess the effect of sleep duration on multiple sites of pain. Statistical analyses were performed using SPSS version 21.0 (IBM, Armonk, NY, USA) and two-sided *P* values of <.05 were considered statistically significant.

## 3 Results

### 3.1 Baseline characteristics

Demographic characteristics of the study subjects are shown in Table 1. Among the 17,108 subjects, 6205 (36.2%) subjects reported musculoskeletal pain in the knee joint (21.8%), hip joint (10.4%), or low back (24.1%). The mean age of subjects with pain was 66.6 ± 0.1 years and 70.4% were women. Among the participants with pain, 27.9% ± 0.7 reported a sleep duration of ≤5 h and 8.9% ± 0.5 participants reported a sleep duration of ≥9 h. Participants with MSK pain were older, female, low family income, less educated, and unpaid employment compared to those without MSK pain (*P* < .001). Participants with MSK pain were male current smokers (*P* = .049), had higher physical activity (*P* < .001), higher BMI (*P* < .001), and high stress level (*P* < .001), and they showed a significantly higher prevalence of osteoarthritis (*P* < .001) and rheumatoid arthritis (*P* < .001). Participants with MSK pain also had longer (≥9 h) and shorter (≤5 h) sleep duration (*P* < .001).

**Table 1:**
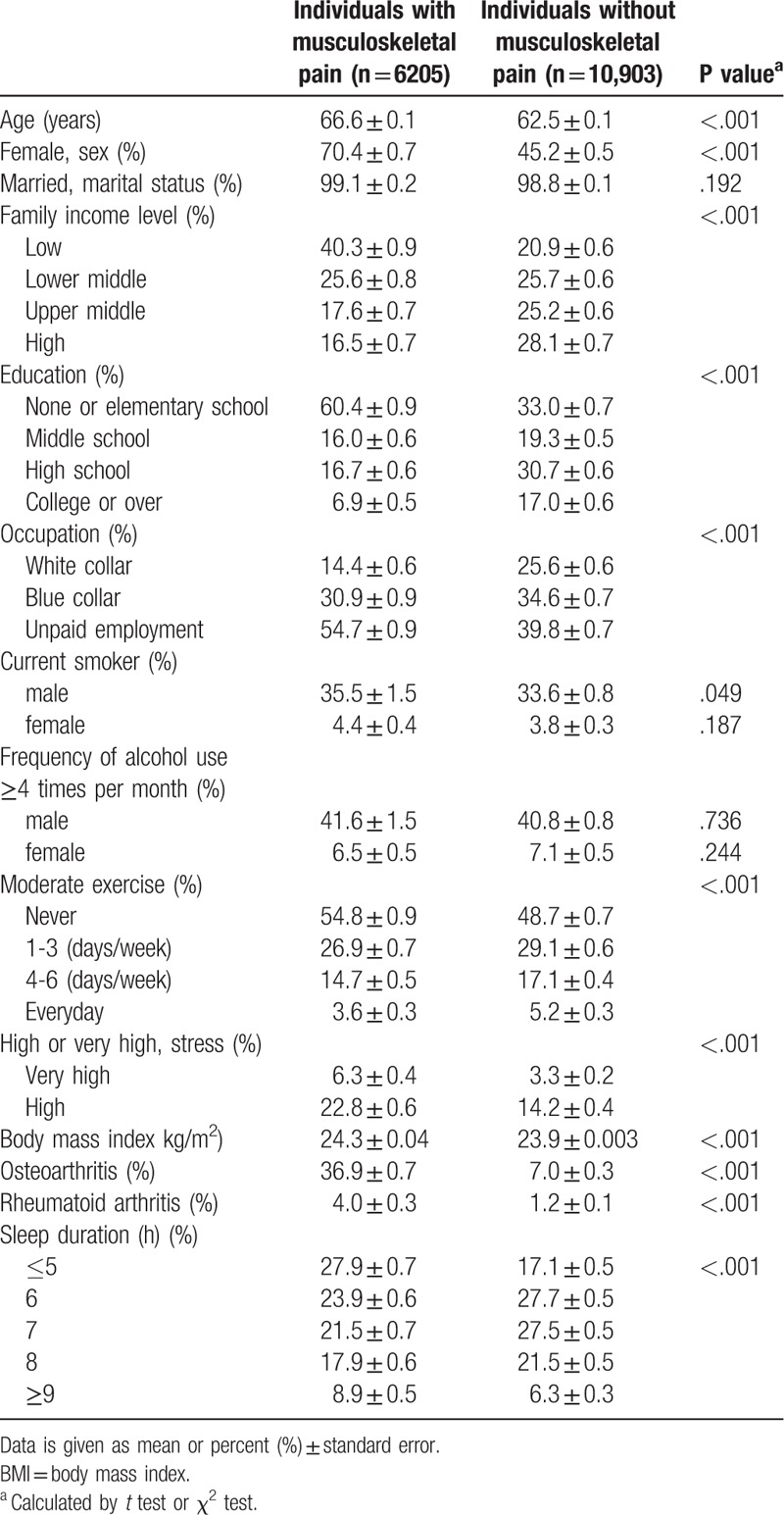
Demographics of the study participants.

### 3.2 Multivariate analyses

**Figure 2:**
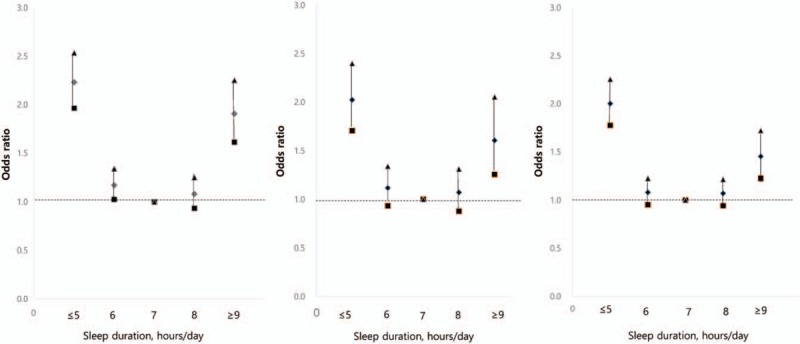
The association between sleep duration and the odds ratio of (a) knee joint pain, (b) hip joint pain, or (c) low back pain, using unadjusted data. Diamonds depict odds ratios for the presence of musculoskeletal pain. Quadrangles represent lower confidence limit triangles represent upper confidence limit.

The association between sleep duration and musculoskeletal pain was examined using multiple logistic regression models (Table 2). Compared with a 7-h sleep duration, which was used as reference, shorter sleep duration (≤5 h) was significantly associated with a high prevalence of knee joint pain (OR = 2.23; 95% CI = 1.96–2.54), hip joint pain (OR = 2.02; 95% CI = 1.71–2.40), and low back pain (OR = 2.00; 95% CI = 1.78–2.25) in unadjusted models. In unadjusted models, longer sleep duration (≥9 h) was also significantly associated with a high prevalence of knee joint pain (OR = 1.91; 95% CI = 1.61–2.25), hip joint pain (OR = 1.61; 95% CI = 1.25–2.05), and low back pain (OR = 1.45; 95% CI = 1.22–1.72) in comparison to 7-h sleep duration. The association between sleep duration and the odds ratio of knee joint pain, hip joint pain, and low back pain using unadjusted data is presented in Figure 2.

**Table 2:**
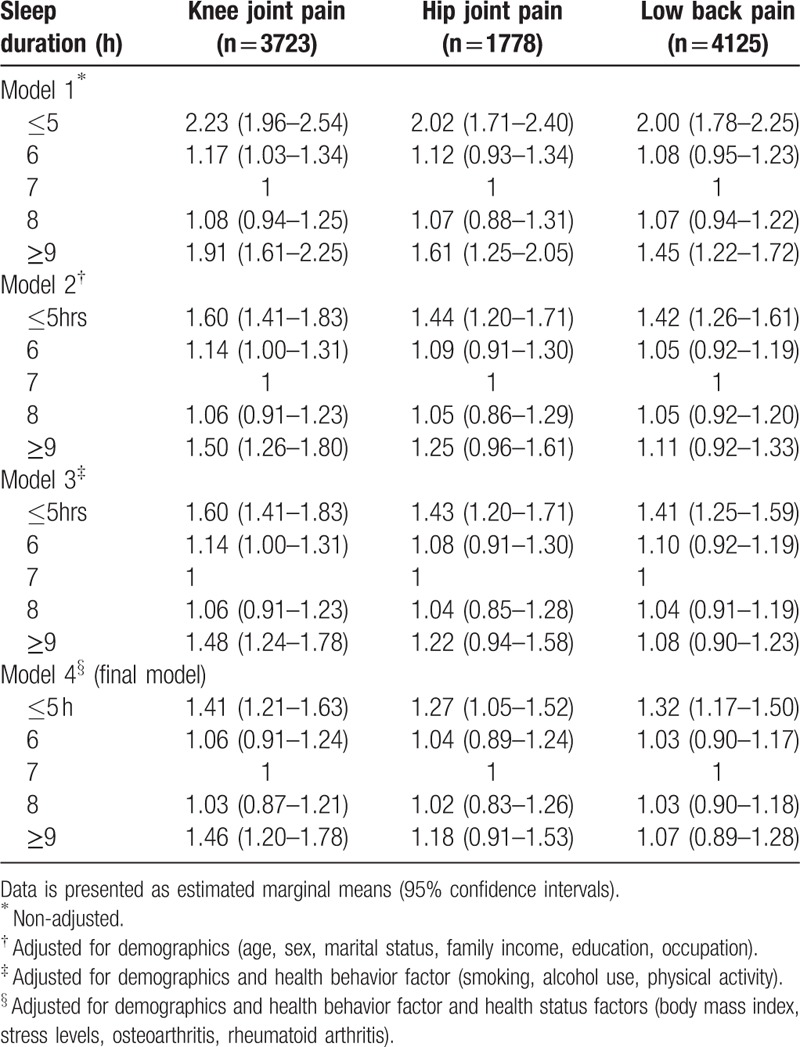
Crude and adjusted odds ratios for sleep duration associated with musculoskeletal pain according to various statistical models.

After adjusting for demographic covariates, a sleep duration of ≤5 h was found to be significantly associated with a high prevalence of knee joint pain (OR = 1.60; 95% CI = 1.41–1.83), hip joint pain (OR = 1.44; 95% CI = 1.20–1.71), and low back pain (OR = 1.42; 95% CI = 1.26–1.61), using 7-h sleep duration as a reference. Longer sleep duration (≥9 h) was also significantly associated with a high prevalence of knee joint pain (OR = 1.50; 95% CI = 1.26–1.80) in model 2. The above-mentioned results were maintained even after adjustments were made in models 3 and 4 (final model).

### 3.3 Dose-response relationship

Finally, we analyzed the association between the number of pain sites and sleep duration. The proportion of multi-site pain (pain in 2 or more anatomical areas) was significantly different between the sleep-duration categories (Table 3). The proportion of those with multi-site pain was significantly higher among those who slept for the shortest duration (≤5 h) or the longest duration (≥9 h), whereas it was lowest among those who slept for 7 h. Sleep duration of ≤5 h (OR = 3.40; 95% CI = 2.66–4.35) and ≥9 h (OR = 2.38; 95% CI = 1.69–3.35) was found to be significantly associated with a high prevalence of joint pain in the 3 areas.

**Table 3:**
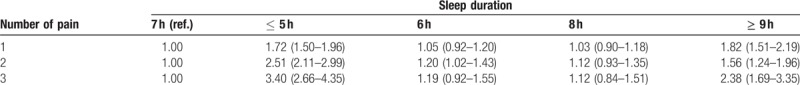
Crude odds ratios for sleep duration associated with number of musculoskeletal pain.

## 4 Discussion

The present study examined the association between self-reported sleep duration and MSK pain in an elderly Korean population using the dataset of the KNHANES. In this study, we confirmed that extreme sleep duration is prevalent in MSK pain subjects and is more prevalent in subjects with multi-site joint pain. As reported in Table 1, participants are more likely to report MSK symptoms if they are older adult, female, less educated, current smokers, with a lower socioeconomic level, higher BMI, lower physical activity, and higher stress level.

Previous studies reported the prevalence of MSK pain in upper extremities, low back, and lower extremities as 62.6%, 72.6%, and 45.7%, respectively, in a Korean population aged 65 years and older.^[15]^ At 67%, the prevalence of MSK pain in China is similar to that in Korea; back pain is the most prevalent form of symptoms.^[16]^ With 59% of United States adults aged 65 and older reporting MSK pain, the prevalence of MSK is higher in Asian than in Western countries.^[17]^ In the present study, 33.6% of older adults aged ≥50 years reported MSK pain in their knee joint (21.8%), hip joint (10.4%), or low back (24.1%). A review of studies regarding MSK symptoms has not determined a consensus on the exact estimates of prevalence, but has reported that these disorders are still rapidly increasing. It necessary to determine the risk factors to ensure the intervention target.^[18,19]^

Our finding, that women have a higher prevalence of reported MSK symptoms, is consistent with previous studies.^[20,21]^ Various studies have suggested explanations such as gender-different pain thresholds, muscle strength, exposure to risks in the work environment, and psychosocial factors.^[22,23]^ Our study indicates that marital status is not associated with the prevalence of MSK symptoms. In a study by Park et al, females without spouses had higher reports of symptomatic osteoarthritis than those with spouses.^[19]^

Consistent with previous research regarding education level, occupation, and family income level, our research suggests that better-educated older Koreans are less likely to experience MSK pain.^[24]^ Higher education has been linked to higher socioeconomic level, which helps individuals not only to acquire a concept of healthy lifestyle but also to influence behaviors that may lead to good health.^[25,26]^ However, more studies are needed to ascertain the reason for the association among socioeconomic level, related lifestyle behaviors, and level of health in the elderly Korean population.

Both our study and previous studies have found that a higher BMI was likely to indicate being overweight or obese, which was associated with MSK pain in the low back and lower extremities.^[1,15,27]^ Notably, studies of obesity and low back pain have reported improvement or resolution in pain or disability with weight loss.^[27–29]^ As obesity rates have increased worldwide, it is reasonable to predict that the prevalence of MSK related to body weight and associated disorders in the population will continue to increase.^[1,30]^ Previous reports evaluating the association between chronic MSK pain and physical exercise have shown conflicting results.^[18,31,32]^ In a large-scale, population-based follow-up study in Norway, physical inactivity was associated with a higher prevalence of chronic widespread MSK pain complaints.^[31]^ Our finding is in accordance with several studies that report a negative correlation between physical exercise and MSK pain.^[31,32]^ In the present study, a subject's exercise level is based on leisure-time exercise only. However, the effect of physical workload in occupational areas might have contributed to the findings. In addition, we could not rule out the potentiality that subjects with MSK pain have not been able to achieve or retain a moderate physical activity level.

Many studies reported that poor psychological states are more prevalent in chronic MSK pain patients and suggested psychological factors are important in the development of MSK pain.^[1,2,33,34]^ Consistent with our result, previous studies demonstrated that stress, anxiety, and depression significantly affect patients’ pain report and pain severity regarding MSK pain.^[1,2,33–35]^

Our research also indicates that subjects with MSK pain are more likely than subjects without MSK pain conditions to smoke, but the association is fairly modest. However, many current articles suggest that smoking is associated with the development and prevalence of back pain^[36,37]^ as well as chronic widespread MSK pain.^[38]^ This last study has also reported a significant dose-response effect of the number of cigarettes smoked daily on the prevalence of low back pain. ^[38]^ However, very few studies have been conducted on the association between alcohol consumption and MSK pain, and research has produced inconsistent results.^[36,38,39]^ Consistent with our result, Yoichi Iizuka suggested that drinking habit is not associated with chronic back pain.^[39]^ However, other research found an association between MSK pain and current drinking habit, or alcohol abuse history before the onset of pain.^[1,40]^

Previous studies investigating the correlation between sleep and MSK pain have reported varying results.^[7,8,9,41]^ These varying results are likely to originate from methodological differences, including choice of target sample, size of sample, measurement method for sleep duration, and adjustments made for various confounding factors. Previous studies have also been limited by small sample sizes and confounding demographic factors.^[2,10,42]^ In the present study, we identified a U-shaped relationship between sleep duration and MSK pain within an adult Korean population. By considering the various confounding factors related to pain and sleep, we were able to increase the strength of our findings. Consistent with our results, in a cross-sectional study of Korean adults, individuals with severe clinical insomnia (OR = 1.112; 95% CI = 1.057–1.170, *P* < .001) reported more frequent severe chronic MSK pain based on data from a single clinical setting.^[2]^ Moreover, using data from the US national health survey, Strine et al found that self-reported insomnia and trouble falling asleep were associated with low back pain (OR = 2.6; 95% CI = 2.4–2.9) and neck pain (OR = 3.5; 95% CI = 3.0–4.0).^[1]^ In a study of Chinese adults, the prevalence of MSK pain was also associated with poor sleep quality.^[23]^ However, few studies have examined the association between long sleep duration and pain in a large general population.

In our study, multi-site musculoskeletal pain, which was experienced in 48.4% of participants, was also associated with both short and long duration of sleep. Kim et al reported that multi-site musculoskeletal pain and pain intensity, including in the low back, lower limbs, and joints, were significantly associated with insomnia in chronic neck pain patients.^[42]^ Li et al also reported that a higher number of painful joints was associated with poor sleep quality, and that 27% of patients with chronic neck pain complained of comorbid musculoskeletal pain, including pain in the low back, lower limbs, and joints.^[41]^ Artner et al reported a higher prevalence of sleep disturbance in a patient subgroup with both chronic neck and back pain than in patients with pain in only one area.^[10]^ Consistent with a previous study, dose-response associations for specific prevalence of MSK pain with sleep problems may increase the chance of experiencing pain in multiple sites within the body.^[43]^

Therefore, the observed association of extreme sleep duration with subsequent increases in multiple pain complaints corresponds well with previous data. Because there is a bi-directional link between pain and sleep, sleep disturbance has often been regarded as a secondary symptom of chronic pain rather than an independent symptom. However, as new findings have emerged, there has been increasing acceptance of indications that insomnia has an independent effect on the development and prognosis of chronic pain.^[7,44,45]^ A micro-longitudinal study has shown that there is a significant curvilinear prospective association between sleep duration and subsequent daily pain complaints (β = −.08; *P* < .0001). In this study, individuals who slept for fewer than 6 h a night, or for 9 h or more, more frequently reported pain the following day. Individuals who slept for 3 h or fewer reported an 81% increase in pain frequency compared to those who slept for 6–9 h; sleeping for more than 11 h was associated with a 137% increase in pain frequency.^[46]^ Additionally, a 28-year follow-up study on industrial employees reported that individuals with sleep disturbances had a 2.1-fold increased risk of back-related hospitalization than those with no sleep disturbances.^[47]^

A number of studies have investigated mechanisms that can explain the link between sleep duration and musculoskeletal pain conditions.^[47,48]^ Laboratory-based sleep studies have suggested that sleep deprivation is accompanied by an increased sensitivity to noxious stimuli and a decrease in endogenous pain-inhibitory processes.^[46–48]^ This may thus explain the observed association between reduced sleep time and increased prevalence of MSK pain. The prospective link between longer sleep duration and elevated MSK pain is more difficult to explain, but it may reflect the effects of an acute condition associated with both lengthened sleep and physical discomfort.^[46]^

The results of our study corroborate previous reports that a long sleep duration is significantly associated with increased inflammation, which also suggests a U-shaped pattern for this association.^[11,49,50]^ A relatively long sleep duration may indicate poor sleep continuity, such as fragmented sleep, which has been associated with enhanced pain perception in some laboratory studies on experimental sleep fragmentation.^[51]^ In the aforementioned study, the total amount of sleep time was less important than the continuity of sleep in shaping next-day pain responses.^[52]^

The present study has several limitations. Firstly, because the KNHANES is a cross-sectional survey, the positive association between many factors and musculoskeletal pain does not imply causality. To identify causal links, longitudinal cohort studies may be required. Secondly, as sleep duration was self-reported, it may be prone to subjective bias; however, previous studies have established that there is generally good agreement between objectively measured sleep and subjectively assessed sleep.^[52,53]^ Thirdly, sleep duration was only investigated over a relatively short period; it is possible that participants’ sleep duration changed during the follow-up period. Finally, the possibility of residual confounding factors due to unmeasured variables or incompletely adjusted covariables cannot be excluded. Despite these limitations, because this study was based on a nationally representative sample, the results could be generalized to a larger population.

In conclusion, an approximately U-shaped relationship was detected between self-reported sleep duration and the presence of musculoskeletal pain in an elderly Korean population. Both longer and shorter sleep durations were associated with a higher prevalence of musculoskeletal pain. Therefore, healthcare professionals who treat patients with MSK pain in routine clinical practice should be aware of the relationship between MSK pain and sleep problems. Specific assessment and treatment of sleep disturbance should be included as an important part of pain management in patients with MSK pain.

## Acknowledgments

The authors thank the subjects whose participation made this study possible.

## Author contributions

**Conceptualization:** Bum-Joo Cho, Sang Ho Yoo, Bumjo Oh, Ju-Seop Kang.

**Data curation:** Sang Ho Yoo, Bumjo Oh.

**Investigation:** Bum-Joo Cho, Sang Ho Yoo, Ju-Seop Kang.

**Methodology:** Bum-Joo Cho.

**Supervision:** Sang Ho Yoo.

**Writing – original draft:** Minyoung Chun, Cholog Yeon.

**Writing – review & editing:** Minyoung Chun.

Minyoung Chun orcid: 0000-0001-7200-5968.
